# Development and validation of prognostic nomogram for T_1-3_N_0_M_0_ non-small cell lung cancer after curative resection

**DOI:** 10.1186/s12885-023-11158-w

**Published:** 2023-07-31

**Authors:** Weijian Mei, Wang Yao, Zhengbo Song, Wenjie Jiao, Lianxin Zhu, Qinghua Huang, Chaolun An, Jianguang Shi, Guiping Yu, Pingli Sun, Yinbin Zhang, Jianfei Shen, Chunwei Xu, Han Yang, Qian Wang, Zhihua Zhu

**Affiliations:** 1 Department of Thoracic Surgery, State Key Laboratory of Oncology in South China, Collaborative Innovation Center for Cancer Medicine, Sun Yat-Sen University Cancer Center, 651 Dongfeng Road East, Guangzhou, 510060 Guangdong China; 2 Department of Interventional Oncology, The First Affiliated Hospital, Sun Yat-Sen University, Guangzhou, China; 3 Department of Medical Oncology, Cancer Hospital of University of Chinese Academy of Sciences; Zhejiang Cancer Hospital, Hangzhou, China; 4 Department of Thoracic Surgery, Affiliated Hospital of Qingdao University, Qingdao, China; 5 Medical College of Nanchang University, Nanchang, China; 6 Queen Mary University of London, London, UK; 7 Department of Breast Surgery, Affiliated Tumor Hospital of Guangxi Medical University, Nanning, China; 8 Department of Thoracic Surgery, Nantong Third People’s Hospital Nantong University, Nantong, China; 9 Department of Thoracic Surgery, Ningbo First Hospital of Zhejiang University, Ningbo, China; 10 Department of Thoracic Surgery, Affiliated Jiangyin Hospital of Southeast University, Jiangyin, China; 11 Department of Pathology Department, The Second Hospital of Jilin University, Changchun, China; 12 Department of Thoracic Surgery, The Second Affiliated Hospital of Medical College, Xi’an Jiaotong University, Xi’an, China; 13 Department of Thoracic Surgery, Taizhou Hospital of Zhejiang Province, Wenzhou Medical University, Taizhou, China; 14 Department of Respiratory Medicine, Affiliated Hospital of Nanjing University of Chinese Medicine, Suqian Hospital of Chinese Medicine, 9 Hongzehu Dong Road, Suqian, 223800 Jiangsu China

**Keywords:** Non-small cell lung cancer, Nomogram, Curative resection

## Abstract

**Background:**

Radical resection plus lymph node dissection is a common treatment for patients with T_1-3_N_0_M_0_ non-small cell lung cancer (NSCLC). Few models predicted the survival outcomes of these patients. This study aimed to developed a nomogram for predicting their overall survival (OS).

**Materials and methods:**

This study involved 3002 patients with T_1-3_N_0_M_0_ NSCLC after curative resection between January 1999 and October 2013. 1525 Patients from Sun Yat-sen University Cancer Center were randomly allocated to training cohort and internal validation cohort in a ratio of 7:3. 1477 patients from ten institutions were recruited as external validation cohort. A nomogram was constructed based on the training cohort and validated by internal and external validation cohort to predict the OS of these patients. The accuracy and practicability were tested by Harrell's C-indexes, calibration plots and decision curve analyses (DCA).

**Results:**

Age, sex, histological classification, pathological T stage, and HI standard were independent factors for OS and were included in our nomogram. The C-index of the nomogram for OS estimates were 0.671 (95% CI, 0.637–0.705),0.632 (95% CI, 0.581–0.683), and 0.645 (95% CI, 0.617–0.673) in the training cohorts, internal validation cohorts, and external validation cohort, respectively. The calibration plots and DCA for predictions of OS were in excellent agreement. An online version of the nomogram was built for convenient clinical practice.

**Conclusions:**

Our nomogram can predict the OS of patients with T_1-3_N_0_M_0_ NSCLC after curative resection. The online version of our nomogram offer opportunities for fast personalized risk stratification and prognosis prediction in clinical practice.

**Supplementary Information:**

The online version contains supplementary material available at 10.1186/s12885-023-11158-w.

## Introduction

Lung cancer is the most significant solid malignancy with high morbidity and mortality worldwide [1, 2]. The current standard of care for patients with T_1-3_N_0_M_0_ non-small cell lung cancer (NSCLC) consists of radical resection plus lymph node dissection or sampling of high quality [3, 4]. The 5-year overall survival (OS) for patients with T_1-3_N_0_M_0_ NSCLC after surgery can reach varied from 50 to 80% [5, 6]. The quality of the surgery, especially the lymph node dissection, would have an effect on the prognosis [7, 8]. Our previous study suggested that the updated hilar and intrapulmonary lymph nodes quantitative standard (HI standard) provide important guidance for pulmonary lymph node dissection and pathological examination in patients with T1–3N0M0 NSCLC [9, 10]. Additionally, previous studies revealed that some factors might have an effect on prognosis, such as the age, sex, and histological classification [11, 12].

Accurate prediction of prognosis at personalized level is important for the decision-making strategy. Among the various prognosis evaluation systems, the tumor, node, metastasis (TNM) staging system is widely considered optimal for differentiating the prognosis of patients with NSCLC [13, 14]. However, TNM staging system only focuses on the tumor size and lymph node involvement but ignores other potential factors such as age, sex and the quality of surgery. Therefore, it is meaningful to build a dedicated clinical prognostic model that includes both TNM staging and other potential variables and thus improve the personalized risk staging system and individual treatment decision making.

Nomogram is a multivariate visualization prediction tool that can incorporates multiple potential factors and thus assess the risk of patients [15, 16]. Several studies have developed nomogram to evaluate the stratified treatment of patients with NSCLC [17–20]. However, to our knowledge, few study establish dedicated nomogram to guide the optimal individual treatment strategy for patients with T1-3N0M0 NSCLC. Therefore, we hypothesize that a newly nomogram consisted of the TNM staging system and other potential factors could supplement to the individual treatment strategy for patients with T1-3N0M0 NSCLC. In this study, we developed a nomogram model in a training cohort from Sun Yat-sen University Cancer Center (SYSUCC) based on T stage, age, sex, histological classification, and HI standard. The models were further validated using an independent internal validation cohort from SYSUCC and an external validation cohort from ten other centers.

## Methods

### Patients’ characteristics

**Fig. 1:**
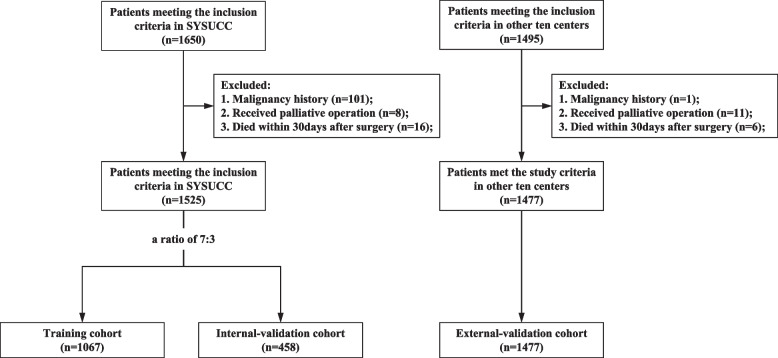
Patient selection scheme for the study

This study was based on the multi-institution registration database of 11 different institutions in China (Sun Yat-sen University Cancer Center; Affiliated Hospital of Qingdao University; Affiliated Jiangyin Hospital of Southeast University; Affiliated Tumor Hospital of Guangxi Medical University; Fujian Cancer Hospital, Fujian Medical University; Ningbo First Hospital, Ningbo Hospital of Zhejiang University; Taizhou First People's Hospital; The Second Affiliated Hospital of Medical College, Xi'an Jiaotong University; The Second Hospital of Jilin University; Third People's Hospital of Nantong City; Zhejiang Cancer Hospital). This study included 3002 patients diagnosed with NSCLC who underwent surgical resection between January 1999 and October 2013. A total of 1525 selected patients from Sun Yat-sen University Cancer Center were randomly divided into training cohort (*n* = 1067) and internal validation cohort (*n* = 458) with a ratio of 7:3. A total of 1477 patients from ten other institutions were included as external validation cohort. The initial patient selection process was shown in Fig. 1. The institutional review board and the ethics committee of Sun Yat-sen University Cancer Center (SYSUCC) approved this retrospective, anonymous analysis of data, and the requirement for written informed consent was waived.

### Definitions

The clinical and pathological characteristics of each NSCLC patient were reviewed retrospectively in the database. The clinical characteristics included age, sex (Male, Female), and laterality (Right, Left). The pathological characteristics included histological classification from the the International Statistical Classification of Diseases and Related Health Problems 10th Revision (Squamous cell carcinoma, Adenocarcinoma, Adenosquamous carcinoma, Others), and pathological T stage from the eighth edition of the AJCC/UICC TNM staging (T1, T2, T3). The HI Standard [9, 10] was defined as recommendation of at least 10 examined lymph nodes, which included at least one station in 10, 11 lymph node and one station in 12, 13, 14 lymph node. Lymph nodes were either dissected in surgical resection or re-sampled by the surgeon after surgery. The final number of lymph nodes was determined by the pathologist.

### Inclusion and exclusion criteria

The patient inclusion criteria were as follows: 1) histologically diagnosed with NSCLC. 2) pathologically staged as T1–3N0M0 according to the AJCC/UICC TNM staging system (eighth edition); 3) with a complete (R0) resection plus lymph node dissection or sampling; 4) with follow-up up to 5 years. The exclusion criteria were as follows: 1) with prior history of metachronous or synchronous malignancy; 2) underwent palliative surgery including sublobectomy, segmentectomy, or wedge-shaped lobectomy; 3)with positive resection margins; 4) with any therapy before surgery (chemotherapy, radiation, target therapy, or any other antitumor therapy); 5) died in 30 days after surgery.

### Follow-up

OS was the only endpoint of our study. OS was defined as the date of surgery to the patient's death or the end of follow-up period. Follow-up data were conducted over the telephone by trained medical staff or staff in the hospital follow-up department. The final follow-up date was October 31, 2018 and all patients were followed up.

### Statistical analysis

Continuous variables such as gender were shown as frequency (%), while categorical variables such as age were shown as median (range).Pearson's chi-square test was used for categorical variables to compare population characteristics, while the independent t-test (or Mann–Whitney U-test) was used for continuous variables. The Kaplan–Meier method was used to estimate the survival and the stratified log-rank test was used to evaluate any survival differences. The univariate and multivariate Cox Proportional Hazard Regression Model was applied to estimate the hazard ratio (HR) and the corresponding 95% confidential interval (CI) for every potential prognostic variable. Significant variables in univariate analyses (*p* < 0.10) would be carried into multivariate analyses. All analyses above were carried out by SPSS 24.0 (SPSS, Chicago, IL) and performed by R version 3.6.0 (The R Foundation for Statistical Computing, Vienna, Austria) through RStudio software (version 1.2.1335). A two-sided level of significance was applied and the p value less than 0.05 indicated statistical significance.

Based on the results of the multivariable analyses, the nomogram was created using the survival and rms packages of R 3.6.0. Discrimination evaluation and calibration evaluation were applied to assess the accuracy of the nomogram. The discrimination evaluation focused on the model’s ability to distinguish patients with different outcomes. Therefore concordance index (C-index) was used as the measuring tool. The calibration evaluation concentrated on how close the predicted probabilities were to the actual outcomes. Calibration plots of the nomogram for 3-, 5- year OS were performed in the training cohorts, internal validation cohorts, and external validation cohort. Decision curve analyses (DCA) was performed to test the reliability of the model and evaluate alternative diagnostic or prognostic tools with superiority.

## Results

### Baseline condition of the study participants

From January 1999 to October 2013, 3002 patients at 11 centers were recruited. A total of 3002 patients were enrolled in the study (see Fig. 1 for the selection process). The clinical and pathological demographics of the patients in the training and validation cohorts were provided in Table 1. The median age was 61.0 (range: 19–83) years. The dominating T stage were T1 and T2, with T1, T2, and T3 cases accounting for 40.5%, 48.5%, and 11.1% of cases. Adenocarcinoma, with proportions of 66.1%, was the main histological type of NSCLC in this study while squamous carcinoma, adenosquamous carcinoma, and other histological types accounted for, 27.9%, 2.9%, and 3.1% of all the participates. Most patients were right lung cancer (58.6%) and less patients meet the HI standard (42.3%). More female (*P* = 0.009) and less patients who meet the HI standard (*P* < 0.001) were found in the external validation cohort when compared with the data of the training and internal validation cohorts. No other significant differences in baseline characteristics was found among the 3 cohorts. All patients had information on survival time with a median follow-up being 65.9 months. The 3- and 5-year OS rates were 93.1% and 85.3% in the training cohort, 93.1% and 84.9% in the internal validation cohorts and 88.1% and 79.8% in the external validation cohort, respectively.

**Table 1:** Baseline characteristics in the study

Characteristic	All (*n* = 3002)	Training cohort (*n* = 1067)	Internal validation cohort (*n* = 458)	External validation cohort (*n* = 1477)
Age (median, range)	61 (19–83)	60 (29–82)	60 (24–80)	61 (19–83)
Sex (n,%)				
Male	1892 (63.0)	692 (64.9)	308 (67.3)	892 (60.4)
Female	1110 (37.0)	375 (35.1)	150 (32.7)	585 (39.6)
Laterality (n, %)				
Right	1758 (58.6)	622 (58.3)	264 (57.6)	872 (59.3)
Left	1244 (41.4)	445 (41.7)	194 (42.4)	605 (40.7)
T stage (n, %)				
1	1215 (40.5)	420 (39.4)	178 (38.9)	617 (41.8)
2	1455 (48.5)	513 (48.1)	233 (50.9)	709 (48.0)
3	332 (11.1)	134 (12.6)	47 (10.3)	151 (10.2)
Meet the HI standard (n, %)				
No	1733 (57.7)	497 (46.6)	228 (49.8)	1088 (73.7)
Yes	1269 (42.3)	570 (53.4)	230 (50.2)	469 (26.3)
Histology (n, %)				
SCC	837 (27.9)	293 (27.5)	127 (27.7)	417 (28.2)
AC	1983 (66.1)	717 (67.2)	308 (67.2)	958 (64.9)
ASC	88 (2.9)	34 (3.2)	13 (2.8)	41 (2.8)
Other	94 (3.1)	23 (2.2)	10 (2.2)	61 (4.1)

*HI standard* hilar and intrapulmonary standard, *SCC* squamous cell carcinoma, *AC* adenocarcinoma, *ASC* adenosquamous carcinoma

### Development nomograms of OS in the training cohort

**Fig. 2:**
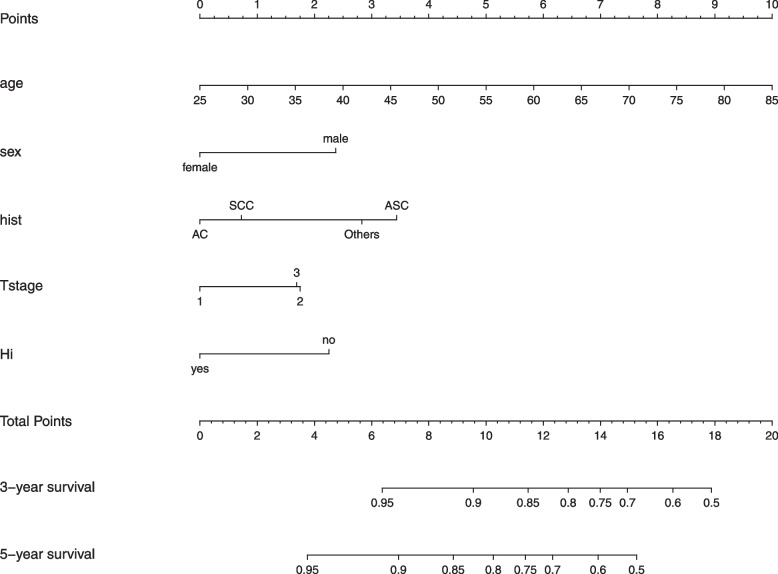
The prognostic nomogram for patients with T_1-3_N_0_M_0_ non-small cell lung cancer after curative resection. Cancer

Univariable and multivariable analyses were performed in the training cohort. On univariate analyses, five factors including age, sex, histological classification, pathological T stage, and HI standard were significantly correlated with OS while laterality was not significantly correlated with OS (*p* = 0.2, see Table 2). These factors were entered into the multivariate analysis, and the results revealed that all the above factors were independent risk factors associated with OS (see Table 2). Based on the five independent risk factors identified in the multivariate analysis, nomogram was developed to predict 3- and 5-year OS for the training cohort (see Fig. 2). Within the five variables that contribute to the nomogram, each variable was assigned a score on the points scale by drawing a vertical line straight down to the axis labeled points. By adding up the scores for each variable and locating it on the total points scale, the individual probabilities of 3- and 5-year OS can be determined.

**Table 2:** Univariate and multivariate Cox analyses of OS for patients in training cohorts

Characteristic	Univariate analysis			Multivariate analysis		
	HR	95% CI	*p*-value	HR	95% CI	*p*-value
Age	1.04	1.03–1.06	< 0.001	1.04	1.02–1.05	< 0.001
Sex						
Male	Reference					
Female	0.46	0.35–0.62	< 0.001	0.59	0.43–0.81	0.001
Laterality						
Right	Reference					
Left	1.19	0.93–1.52	0.2	1.13	0.88–1.45	0.3
Tstage						
1	Reference					
2	1.40	1.07–1.85	0.015	1.48	1.12–1.95	0.006
3	1.75	1.21–2.54	0.003	1.46	0.99–2.15	0.055
Meet the HI standard						
No	Reference					
Yes	0.59	0.46–0.76	< 0.001	0.60	0.47–0.78	< 0.001
Histology						
SCC	Reference					
AC	0.65	0.50–0.85	0.001	0.85	0.64–1.13	0.3
ASC	1.65	0.97–2.82	0.066	1.84	1.07–3.17	0.028
Other	1.14	0.50–2.62	0.7	1.63	0.71–3.76	0.3

*HR* Hazard Ratio, *CI* Confidence Interval, *HI standard* hilar and intrapulmonary standard, *SCC* squamous cell carcinoma, *AC* adenocarcinoma, *ASC* adenosquamous carcinoma

### Predictive performance of the nomograms

**Fig. 3:**
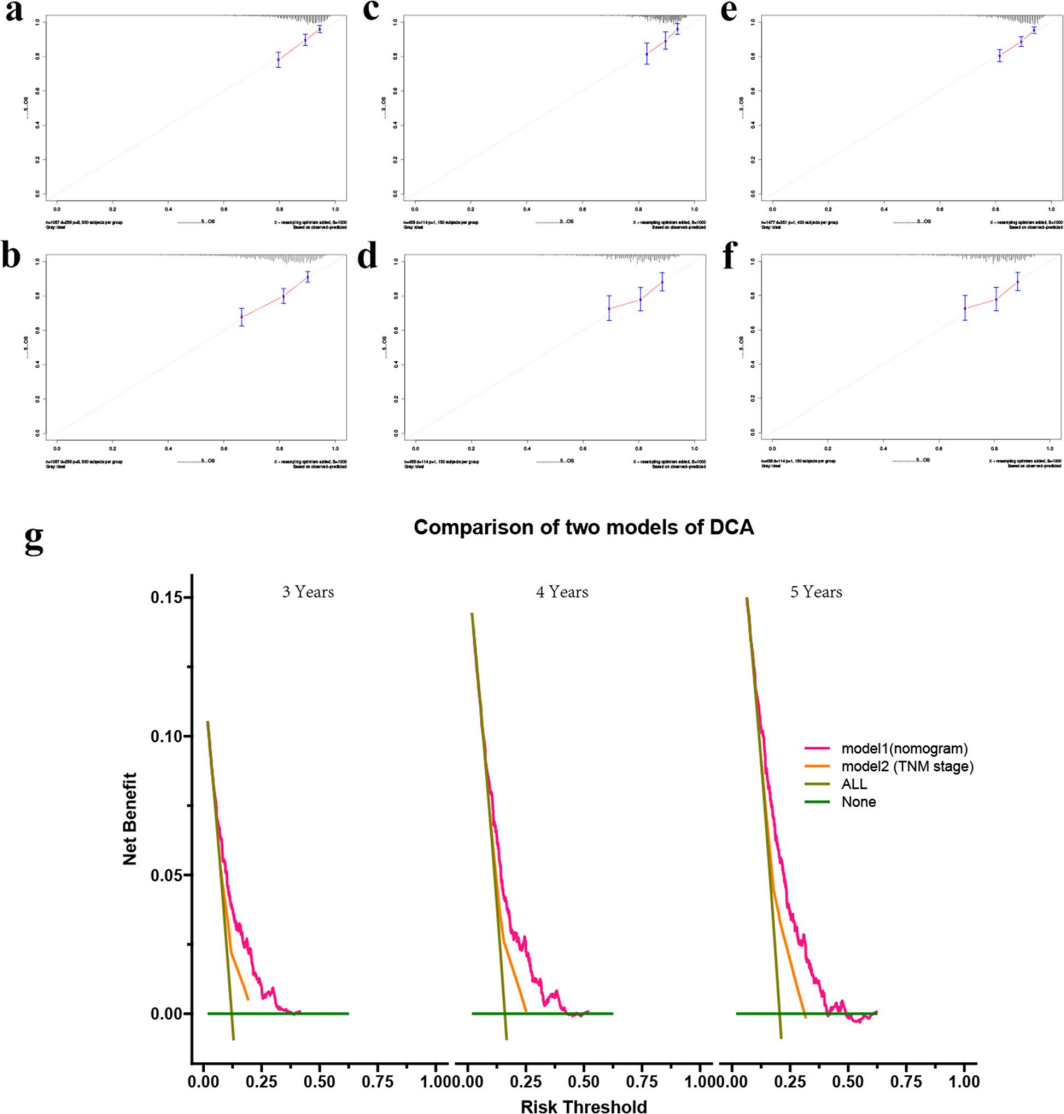
The calibration plots (**a**-**f**) for predicting OS probability at 3 years, and 5 years in three cohorts and the DCA curves (**g**) of the nomogram and TNM staging system for 3-years, 4-years, and 5-years

The predictive performance of the traditional TNM staging system and our nomogram were examined. The traditional TNM staging system showed unsatisfied discriminative ability (training cohort: C index, 0.545; 95% CI, 0.508–0.582; internal validation cohort: C index, 0.517; 95% CI, 0.462–0.572; external validation cohort: C index, 0.602; 95% CI, 0.571–0.633). The C indices of the nomogram for OS estimates were 0.671 (95% CI, 0.637–0.705),0.632 (95% CI, 0.581–0.683), and 0.645 (95% CI, 0.617–0.673) in the training cohorts, internal validation cohorts, and external validation cohort, which demonstrated a good level of discriminative ability. As the calibration curves showed, there was an excellent agreement between the predicted OS by the nomogram and the actual OS probabilities we observed in the training cohorts, internal validation cohorts, and external validation cohort (see Fig. 3a-f). In addition, DCA curve analysis was performed on nomogram and TNM staging system, and found that the performance of the nomogram for OS was better than that of the TNM staging system (see Fig. 3g).

### Development of online version of our nomogram

An online version of our nomogram can be accessed at https://sysucc123456.shinyapps.io/nomograme/ for the researchers and clinicians who are interested in our study. With the help of the online nomogram, individual predicted survival probability across time can be determined by inputting the corresponding factors and reading the individual output figures and tables derived from the webserver. Clinicians can easily assess the difference of the survival probability considering whether the surgery meet the HI standard or not. For example, a 60 year-old male with pT3N0M0 lung squamous cell carcinoma received curative resection, his 5-year survival probability would drop from 0.780 (95% CI, 0.720–0.860) if the surgery meet the HI standard to 0.670 (95% CI, 0.580–0.770) if the surgery failed to meet the HI standard. The predicted survival probability and the Kaplan–Meier curve of estimated OS for her were shown in the Figure S1 and Figure S2.

## Discussions

It is important to choose suitable strategy at personalized level on the basis of accurate prediction of prognosis. Considering that the TNM staging system only takes the tumor size and lymph node involvement into considerations, a dedicated nomograms with more prognostic factors may be more accurate in predicting survival than the TNM staging system [14]. Although several prognostic models have been developed for lung cancer [17–20], a dedicated nomogram has not been established for patients with T1-3N0M0 NSCLC after curative resection. Therefore, we explored the clinical characteristics, pathological characteristics, and prognosis of these patients via a Chinese multi-institutional retrospective database. Most significantly, we established a comprehensive nomogram based on 5 optimal prognostic variables to predict the survival probability of patients with T1-3N0M0 NSCLC after curative resection. A series of validations were done to evaluate the predictive ability and clinical utility of our nomogram. Finally, the nomogram was confirmed to display better discriminatory power in the prediction of survival than the TNM staging system. Additionally, an online version of our nomogram was established as a rapid and user-friendly adjunct tool for clinicians for clinicians to weigh the risks and benefits of more aggressive or more conservative anticancer therapies.

In this large population study, six variables including age, sex, laterality, histological classification, pathological T stage, and HI Standard were reviewed [10]. We identified that five of six factors, except laterality, were correlated with OS in univariate and multivariate analysis. Most of these factors were consistent with previous findings on risk factors for NSCLC [9, 10, 17–20]. However, the correction between laterality and survival varied from different studies [19, 21–23]. Considering its statistical insignificance in our study, we decided not to identified it as variables used in the nomogram. HI Standard, a newly developed standard for lymph node dissection for patients with T1-3N0M0 NSCLC in our previous study, had a considerable impact on survival in univariate and multivariate analysis, which confirmed the importance of sufficient lymph node dissection [10]. Ultimately, we identified age, sex, histological classification, pathological T stage, and HI Standard as variables used in the nomogram.

To our knowledge, this is the first dedicated nomogram to predict OS based on the combination of the TNM staging system and other potential prognostic factors, which may benefit the optimal stratified treatment regimens in patients with T1-3N0M0 NSCLC. Accuracy of the nomogram was assessed by discrimination evaluation as well as calibration evaluation. Our nomogram showed perfect discriminative ability (training cohort: C index, 0.671; 95% CI, 0.637–0.705; internal validation cohort: C index, 0.632; 95% CI, 0.581–0.683; external validation cohort: C index, 0.645; 95% CI, 0.617–0.673). Besides, our nomogram showed excellent agreement in calibration plots for 3-/5- year OS of training cohorts, internal validation cohorts, and external validation cohort. DCA curve was performed to ascertain the clinical usefulness of the nomogram and showed good clinical applicability of the nomogram in predicting 3- and 5-year OS of the patients. All the evaluation above confirmed that our nomogram was an excellent model with a powerful prognostic performance to predict OS in patients with T1-3N0M0 NSCLC after curative resection.

In our study, 3002 patients at 11 different centers in China were chosen, which is much larger than previous studies concerning the nomogram of T1-3N0M0 NSCLC. In order to simplify the application process and facilitate decision-making, we developed an online version at https://sysucc123456.shinyapps.io/nomograme/. For clinical application, clinicians could input corresponding predictors online directly anytime and anywhere to obtain an individual’s survival probability with 95% CI, and then give patients individualized suggestion on subsequent adjuvant therapy as well as the strategy of follow-up management. More importantly, the nomogram showed the difference of the survival probability considering whether the surgery meet the HI standard or not. This highlighted the importance of pulmonary lymph node dissection and pathological examination in patients with T_1-3_N_0_M_0_ NSCLC. It is worth mentioning that this study did not include patients who received sublobectomy, segmentectomy, or wedge-shaped lobectomy as the role of these kind of surgery plays in NSCLC is controversial for a long time [24–27].

Although our nomogram showed excellent discrimination and performance, our study still has several limitations. First, considering the retrospective nature of this study, a selection bias might be inevitable although we have included relatively large training, internal validation, and multi-center external validation cohorts to construct the nomogram, which may reduce the bias caused by the retrospective data analysis. Second, our nomograms are based on Chinese population and therefore may not be generalizable to different patient populations in other country. Third, our database only contained some general information but lack more potential factors, such as information about the postoperative adjuvant therapy, which may cause bias in our study. Given these limitations above, our nomogram still need further validation in prospective clinical trials or other patient population. Despite these limitations, our dedicated nomogram represents an important and effective tool to estimate the prognosis of an eligible patient, and thus offering more advice on personalized therapy strategy and follow-up management.

## Conclusions

In summary, we develop and validate a nomogram that showed good accuracy and reliability to predict 3-year and 5-year OS of patients with T1-3N0M0 NSCLC after curative resection. The online version of our nomogram offer opportunities for fast personalized risk stratification and prognosis prediction in clinical practice.

## Supplementary Information

**Additional file 1: Figure S1.** The predicted 5-year survival probability for a 60 year-old male with pT3N0M0 lung squamous cell carcinoma received curative resection.** Figure S2.** The predicted survival probability of OS for a 60 year-old male with pT3N0M0 lung squamous cell carcinoma received curative resection.

## Data Availability

The study data will be available following publication to researchers wishing to do meta-analyses or for other research proposals, subject to approval and agreement from the study sponsor (Zhihua Zhu). Requests should be made to zhuzhh@sysucc.org.cn.
