# Supplementary material for: Development and validation of prognostic nomogram for T1-3N0M0 non-small cell lung cancer after curative resection
Source: BMC Cancer. 2023 Jul 31;23:715. doi: 10.1186/s12885-023-11158-w (PMC10391852; doi:10.1186/s12885-023-11158-w)

Supplementary materials for

# Development and validation of prognostic nomogram for T_1-3_N_0_M_0_ non-small cell lung cancer after curative resection

**Table of Contents**

[Figure S1. The predicted 5-year survival probability for a 60 year-old male with pT3N0M0 lung squamous cell carcinoma received curative resection. 3](#_Toc1021742959)

[Figure S2. The predicted survival probability of OS for a 60 year-old male with pT3N0M0 lung squamous cell carcinoma received curative resection. 4](#_Toc1154670501)

# Figure S1. The predicted 5-year survival probability for a 60 year-old male with pT3N0M0 lung squamous cell carcinoma received curative resection.

His survival probability would drop from 0.780 (95% CI, 0.720-0.860) if the surgery meet the HI standard (blue line) to 0.670 (95% CI, 0.580-0.770) if the surgery failed to meet the HI standard (black line).
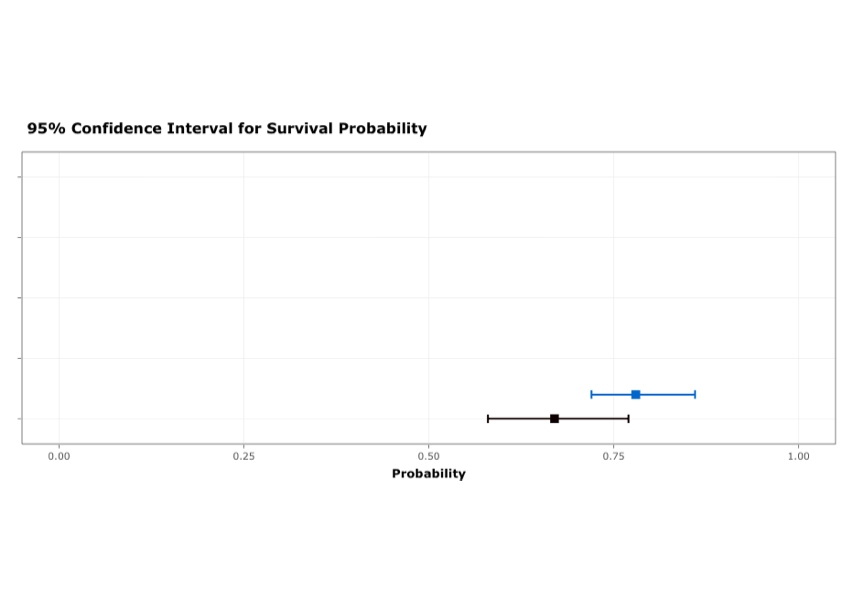


# Figure S2. The predicted survival probability of OS for a 60 year-old male with pT3N0M0 lung squamous cell carcinoma received curative resection.

His survival probability would would be better if the surgery meet the HI standard (blue line) than if the surgery failed to meet the HI standard (black line). The follow up time was shown by month.


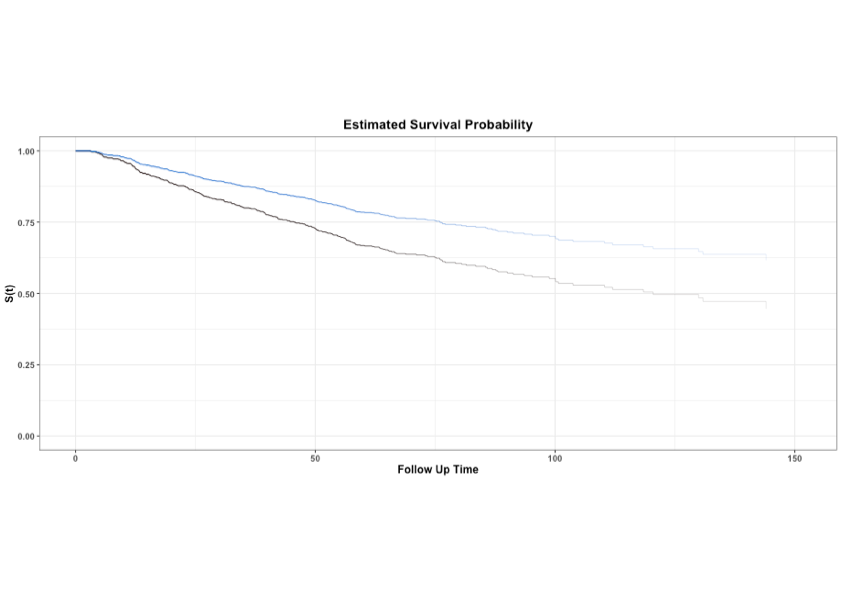

Supplement: Supplementary file 1 — Additional file 1: Figure S1. The predicted 5-year survival probability for a 60 year-old male with pT3N0M0 lung squamous cell carcinoma received curative resection. Figure S2. The predicted survival probability of OS for a 60 year-old male with pT3N0M0 lung squamous cell carcinoma received curative resection. [file 12885_2023_11158_MOESM1_ESM.docx]
